# Altitude, latitude and climate zone as determinants of mountain hare (*Lepus timidus*) coat colour change

**DOI:** 10.1002/ece3.10548

**Published:** 2023-10-01

**Authors:** Allan W. Stokes, Tim R. Hofmeester, Neri H. Thorsen, John Odden, John D. C. Linnell, Simen Pedersen

**Affiliations:** ^1^ Faculty of Applied Ecology, Agricultural Sciences and Biotechnology Inland Norway University of Applied Sciences Koppang Norway; ^2^ Department of Wildlife, Fish, and Environmental Studies Swedish University of Agricultural Sciences Umeå Sweden; ^3^ Norwegian Institute for Nature Research Oslo Norway; ^4^ Norwegian Institute for Nature Research Lillehammer Norway

**Keywords:** lagomorph, moult, phenology, phenotypic trait, subarctic

## Abstract

Local adaptation to annually changing environments has evolved in numerous species. Seasonal coat colour change is an adaptation that has evolved in multiple mammal and bird species occupying areas that experience seasonal snow cover. It has a critical impact on fitness as predation risk may increase when an individual is mismatched against its habitat's background colour. In this paper, we investigate the correlation between landscape covariates and moult timing in a native winter‐adapted herbivore, the mountain hare (*Lepus timidus*), throughout Norway. Data was collected between 2011 and 2019 at 678 camera trap locations deployed across an environmental gradient. Based on this data, we created a Bayesian multinomial logistic regression model that quantified the correlations between landscape covariates and coat colour phenology and analysed among season and year moult timing variation. Our results demonstrate that mountain hare moult timing is strongly correlated with altitude and latitude with hares that live at higher latitudes and altitudes keeping their winter white coats for longer than their conspecifics that inhabit lower latitudes and altitudes. Moult timing was also weakly correlated with climate zone with hares that live in coastal climates keeping their winter white coats for longer than hares that live in continental climates. We found evidence of some among year moult timing variation in spring, but not in autumn. We conclude that mountain hare moult timing has adapted to local environmental conditions throughout Norway.

## Graphical abstract

We used a nationwide camera trap network to provide the first analysis of how mountain hare (*Lepus timidus*) moult timing is correlated with environmental variables across a large geographic area spanning 1300 km. Our results demonstrate that mountain hare moult timing is strongly correlated with altitude and latitude with hares that live at higher latitudes and altitudes keeping their winter white coats for longer than their conspecifics that inhabit lower latitudes and altitudes. We also found some evidence of among year phenotypic variation in spring.

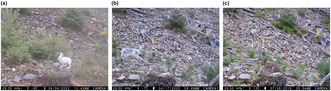

## 1 INTRODUCTION

For species living in seasonal environments (e.g. from summer to winter or from dry to wet season) local adaptations to annually changing environmental conditions may evolve. Numerous species have evolved to time their life history events to match these changes in local seasonal conditions (Bradshaw & Holzapfel, 2007; Williams et al., 2015). To time these phenological events, one reliable ‘zeitgeber’ is daylength or photoperiod (Gwinner, 2003; Hofman, 2004), either on its own or in combination with other variables, such as temperature (Jackes & Watson, 1975; Larkin et al., 2001; Watson, 1963) and snow cover (Flux, 1970; Watson, 1963; Zimova et al., 2014).

Many animal species use photoperiod to time breeding (Coppack & Pulido, 2004; Dawson et al., 2001; Goldman, 1991; Gwinner, 1996b), moulting (Bissonnette & Bailey, 1944; Lesher & Kendeigh, 1941; Lyman, 1943), migration (Gwinner, 1996a) and other life history events. As photoperiod remains constant between years at specific locations, between year variation in local conditions could result in photoperiod timed phenological events being mistimed against the local environment. Fluctuations in environmental variables, such as precipitation (Villellas et al., 2014) and temperature (Ashmore & Janzen, 2003; Kreyling et al., 2019), can result in increased within‐species phenotypic variation in a variety of plant and animal species when compared to individuals of the same species that live in more stable habitats. Consequently, synchrony in phenological timing of individuals within a population is expected to increase with climate stability.

Animals occupying areas that are seasonally covered by snow live in environments that change from dark in summer to white in winter. As a predator avoidance strategy, at least 21 species have adapted seasonal changes in colouration of fur and feather (Mills et al., 2018; Zimova et al., 2018), which provides camouflage in both a winter white and summer dark landscapes (Cott, 1940; Merilaita & Lind, 2005; Wallace, 1879). To provide optimal camouflage, the timing of coat colour change should be synchronised with the period of continuous snow cover. Mismatched timing of coat colour change is linked to range contractions and population declines in several species including snowshoe hares (*Lepus americanus*) (Diefenbach et al., 2016; Sultaire et al., 2016), mountain hares (*Lepus timidus*) (Acevedo et al., 2012; Pedersen et al., 2017), rock ptarmigan (*Lagopus muta*) (Imperio et al., 2013) and white‐tailed ptarmigan (*Lagopus leucura*) (Wang et al., 2002), showing the importance of synchronising moult timing to habitat conditions. However, snow conditions might not be stable from year to year, and there might be seasonal differences in the predictability of the appearance and disappearance of snow. In autumn, there are usually multiple snowfalls with interspersed thawing events that completely remove snow cover. In spring, snow disappearance is more likely to be permanent until the following autumn. Therefore, snow cover is likely to be more stable in spring compared to autumn.

Snowshoe hares that occupied higher altitudes and latitudes expressed winter coats for a longer time than their low latitude (Grange, 1932, but see Zimova et al., 2019) and altitude conspecifics (Holmgren et al., 2001; Nowak et al., 2020; Zimova, Giery, et al., 2020). Also, increased snow cover in continental areas is likely to result in hares living in these areas keeping their winter coats for longer than hares in coastal areas. Snowshoe hares (Zimova et al., 2014) and least weasels (*Mustela nivalis nivalis*; Atmeh et al., 2018) exhibit some phenotypic variation in moult timing in parts of their distribution during the spring moult, when transitioning from white to brown, but not during the autumn moult when transitioning from brown to white. Therefore, seasonal coat colour change is expected to be a more synchronised process in spring, in both start and end date, compared to autumn. This could result in increased among year variation in spring moult timing compared to autumn moult timing. This is the first long‐term study over a geographical area large enough to test these predictions.

Mountain hares express seasonal coat colour change in most of their range, except the subspecies (*L. t. hibernicus*) found in Ireland (Mills et al., 2018). It is a generalist herbivore inhabiting boreal and alpine areas that occupy a wide range of climatic, latitudinal and altitudinal gradients, experiencing large variations in winter snow cover duration. They have a circumpolar distribution spread across Europe and Asia from Britain, Ireland and the Faroe Islands in the west to Japan in the east (Angerbjorn & Flux, 1995). They are native to Norway and are found throughout the country with their distribution ranging from sea level to around 1600 m, which is above the tree line. Coastal areas in the south and south‐west of Norway experience relatively short snow cover duration compared to inland areas and areas in the north (Schuler et al., 2006) with coastal areas in the south and south‐west receiving as little as 1 month of snow cover per year (Tallaksen et al., 2018). Additionally, coastal areas experience greater between‐year variation in the depth and extent of snow cover than inland areas (www.senorge.no).

Here we provide the first quantitative study of mountain hare moult timing variation using 9 years of data collected at 678 camera locations along an extensive geographic gradient in Norway. Proximate factors such as temperature, snow cover and forest cover may affect moult timing. However, in this first attempt to explain moult timing variation in Norwegian mountain hares, we focused on large‐scale geographic gradients in altitude, latitude and climate, which may be the ultimate causes of moult timing variation. We utilised a Bayesian multinomial logistic regression model to study (1) how moult timing varied along broad‐scale geographical gradients in altitude, latitude and climate and (2) how moult timing varied among years and seasons. First, we used altitude, latitude and climatic zone, distinguishing between coastal and continental climates, as indicators of local geographical conditions we predicted that hares living at high altitudes and latitudes and in continental climates would keep their winter white coats for longer than their conspecifics at low altitudes and latitudes and in coastal climates. Second, based on previous snowshoe hare and least weasel studies, we predicted that moult timing would be more synchronised among individuals in spring, resulting in reduced population‐level variation in moult start and end, compared to autumn. Third, we predicted larger among year variation in the timing of moult in spring compared to autumn.

## 2 METHODS

### 2.1 Data collection

**FIGURE 1:**
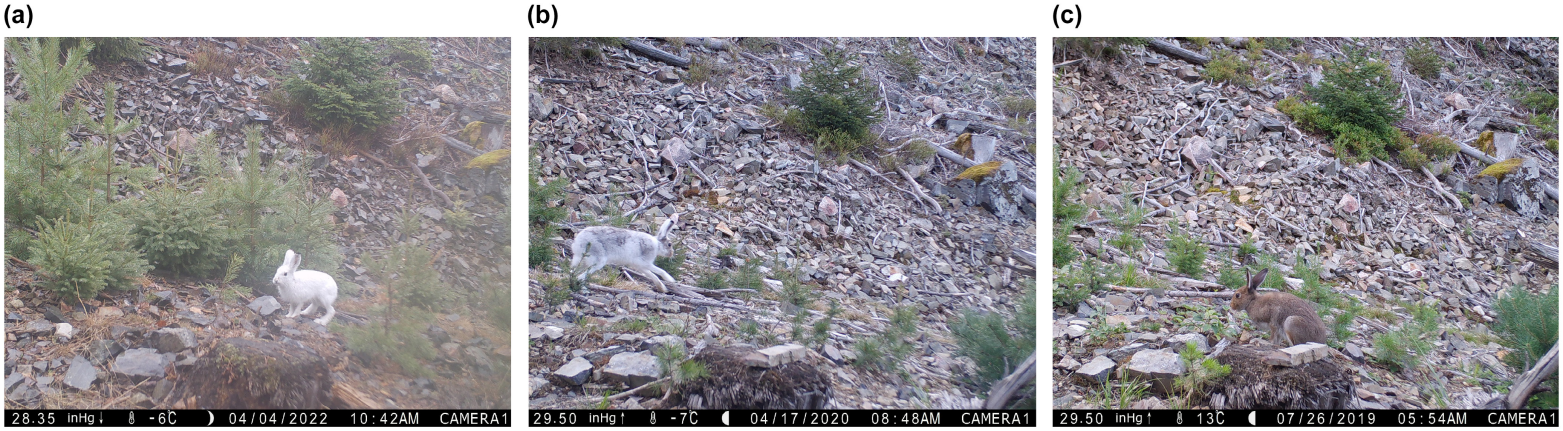
Mountain hares in (a) white, (b) moulting and (c) brown coat colour stage captured by camera traps deployed in Norway by the SCANDCAM project (© NINA).

**FIGURE 2:**
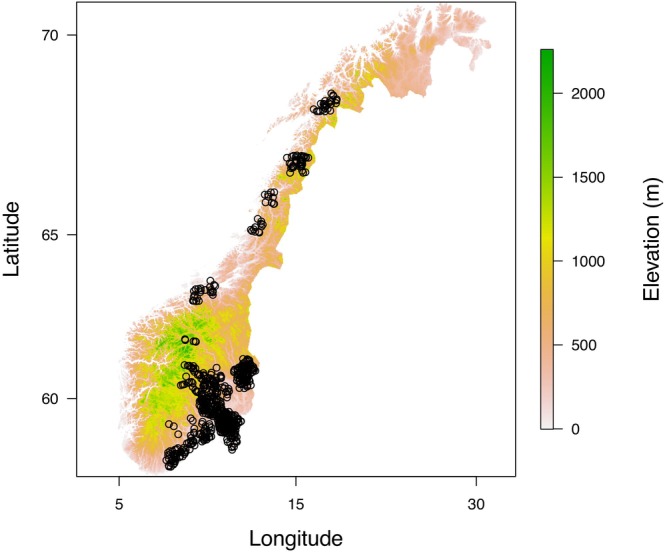
The location of 678 camera trap sites that recorded a mountain hare observation during 2011–2019 (black circles). The camera traps were deployed across an environmental gradient within Norway by the SCANDCAM project, with latitudes varying between 58° N and 69° N and altitudes varying between 0 and 841 m above sea level.

We utilised images from camera traps (Figure 1) that were deployed by the SCANDCAM project (www.viltkamera.nina.no) to monitor the Eurasian lynx (*Lynx lynx*) (Hofmeester et al., 2021). Camera traps were deployed in multiple study areas in an extensive grid with approximately one camera per 50 km^2^ grid cell (Figure 2). We selected all mountain hare records from the period between 10 January 2011 and 25 June 2019. Images containing mountain hares were recorded at 678 locations across Norway (Figure 2), spanning a latitudinal gradient from 58° N to 69° N and altitudes from 0 to 841 m above sea level and climate zone PCA values were between −2.68 (coastal climate) and 2.86 (continental climate). To reduce pseudo‐replication, we discarded observations recorded within 60 min of the previous observation. Mountain hares and invasive European hares (*Lepus europaeus*), which are also present in south‐eastern Norway (Viken County), were differentiated using the species descriptions contained in Smith et al. (2018). When mountain hares were identified, we estimated the proportion of the hares' coat (excluding the long white belly) which was white. We classified moulting stage into three categories modified from Zimova, Barnard, et al. (2020). (1) Hares with ≥90% white fur were classified as ‘white’, (2) hares with ≤10% white fur were classified as ‘brown’ and (3) all other hares were classified as ‘moulting’. All images were accessed on www.viltkamera.nina.no and were visually classified by one of two observers. They were quality‐controlled whenever the observer was uncertain of the classification.

### 2.2 Covariates

We divided the year into two seasons, ‘spring’ and ‘autumn’. Spring was defined as ordinal days 1–212 (1 January to 31 July in non‐leap years) and autumn for the rest of the year. Ordinal day 212 was chosen as all mountain hares had moulted to their summer brown coats by this date and had not started moulting back to winter white. Altitude and latitude were extracted based on camera trap positions. We obtained altitude from a digital elevation model (DEM) with 50 m^2^ resolution (Korsnes, 2018) (Appendix S1a) using the *raster* package's (Hijmans, 2022) extract function. We obtained climate zone as a continuous variable with a resolution of 1 km^2^ from Bakkestuen et al. (2008) (Appendix S1b). We converted climate zone vector data to a raster using the *fasterize* (version 1.0.4) package (Ross, 2020). Bakkestuen et al. (2008) mapped climate zones by conducting principal component analysis (PCA) using terrain data, climatic data, hydrological data and geological data. A positive PCA value indicates a continental climate whereas a negative value indicates a coastal climate.

### 2.3 Data analysis

We created two models, one for spring and one for autumn. We used multinomial logistic regression to estimate the probability of a hare being in moult category *i* (white, moulting or brown) on each ordinal day *d*. We included year *k*‐specific intercepts to test if moult timing varied between years. The model used the following equation:

$$p\left(y=i\right)=\frac{e^{\alpha_{\mathrm{ik}}+\beta_{1i}\times d+\beta_{2i}\times a_{j}+\beta_{3i}\times l_{j}+\beta_{4i}\times c_{j}+s_{\mathrm{ij}}}}{1+\sum_{m=1}^{i-1}e^{\alpha_{i}+\beta_{1i}\times d+\beta_{2i}\times a_{j}+\beta_{3i}\times l_{j}+\beta_{4i}\times c_{j}+s_{\mathrm{ij}}}}$$

where *d* is ordinal day, and *a*_*j*_, *l*_*j*_ and *c*_*j*_, respectively, are the altitude, latitude and climate zone at camera site *j* (as adapted from Zimova, Giery, et al., 2020; Zimova et al., 2019). Parameters *β*_1–4*j*_ represent the slopes for the different covariates. We included a category *i* and year *k* specific intercept *α*_*ik*_ as well as a site‐specific random intercept *s*_*ij*_ to account for repeated observations per camera site. The brown category was set to 0 in both spring and autumn models to provide a baseline for comparison with the moulting and white categories.

We implemented the models in a Bayesian framework using JAGS (Denwood, 2016) called from R V4.1.3 (R Core Team, 2022) with the *jagsUI* package (version 1.5.2) (Kellner, 2021). We standardised altitude, latitude and climate zone (mean = 0, SD = 1) before running the models. Ordinal day was included as an explanatory covariate to enable estimation of the probability of a hare being white, brown or moulting between coats on specific days of the year. We checked for collinearity between the covariates using both the variance inflation factor (VIF) and Pearson correlation coefficient. For every covariate combination, the Pearson values were below 0.6 (Appendix S2) and VIF values were below 2.0 (Appendix S3).

We used uninformative, normally distributed priors with a mean of 0 and precision of 0.01 for all slopes and the year *k*‐specific intercepts. For the site *s* random intercept, we used a mean of 0 and a standard deviation defined as a vague prior with a uniform distribution between 0 and 100. We ran the models with three chains all thinned by 100 for 120,000 iterations, with a burn‐in of 60,000 iterations. We confirmed model convergence using traceplots and the Gelman‐Rubin convergence statistic (R‐hat) (Brooks & Gelman, 1998) with all variables used in the final models having R‐hat values of 1.10 or less. Additional models, in which the dataset was subset to only include cameras located south of 61° N, were used to test if model performance was affected by camera trap placement north of this latitude. The results were consistent with those obtained using the full dataset indicating that camera placement north of 61° did not affect model performance.

**FIGURE 3:**
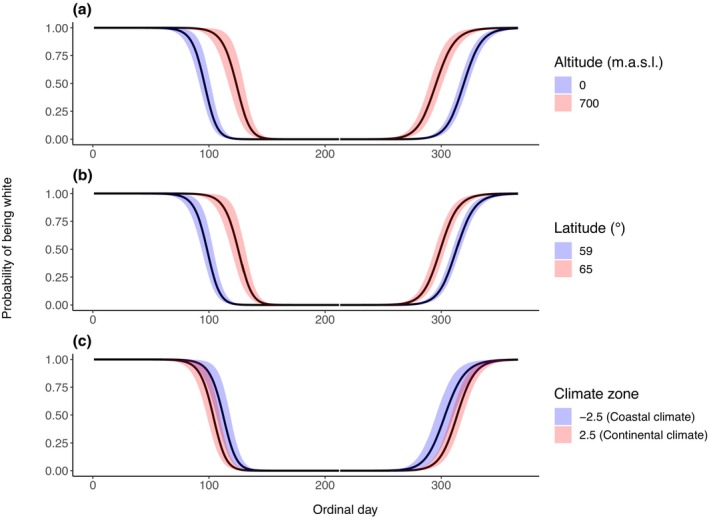
Timing of spring and autumn moult giving the probability of mountain hares being white compared to the combined probability of being brown or moulting at different altitudes (a), latitudes (b) and climate zones (c). Solid lines represent the predicted probability of being white and shaded areas indicate the 95% credible intervals. Figures use the mean of all intercepts included in the final models. The explanatory variables not plotted in each graph were set to the mean value.

**FIGURE 4:**
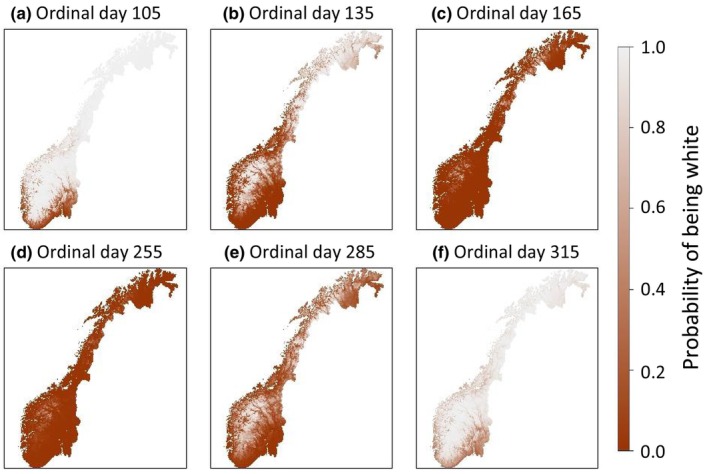
Prediction maps with a resolution of 1 × 1 km^2^ giving the probability of mountain hares being white compared to the combined probability of being brown or moulting across Norway on ordinal days (a) 105 (15th April), (b) 135 (15th May), (c) 165 (14th June), (d) 255 (12th September), (e) 285 (12th October) and (f) 315 (11th November). The probability of being white was predicted using the model output and the environmental covariates contained in every cell. See Appendix S6 for an animated map containing every ordinal day.

**FIGURE 5:**
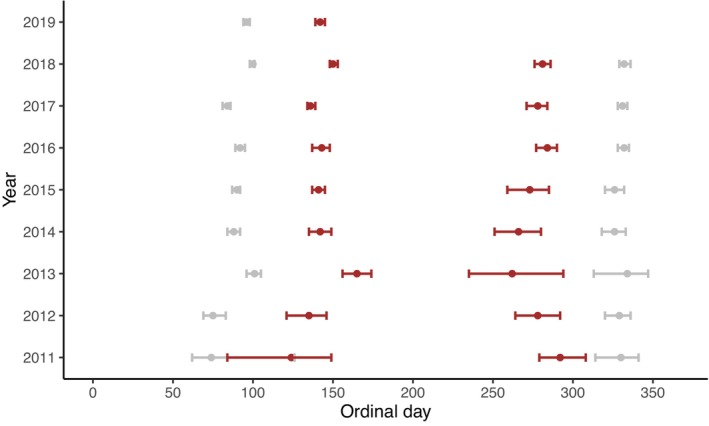
Between year intercepts with 95% CI for spring and autumn at mean altitude (257 m), latitude (61° N) and climatic zone. Grey dots with 95% CI represent the mean ordinal day on which 90% of hares are white (10% brown or moulting). Brown dots with 95% CI represent the mean ordinal day on which 90% of hares are brown (10% white or moulting). The supplemental information contains sample sizes (Appendix S4), number of days between the midpoints (Appendix S7) and intercept beta values (Appendix S8).

We produced all figures using the *ggplot2* (Wickham, 2016), *raster* (Hijmans, 2022) and *cowplot* (Wilke, 2020) packages. When plotting the correlation between the explanatory variables and moult timing (Figures 3 and 5), the variables not being manipulated were set to the mean value. Figures 3 and 4 give the probability of being white compared to the combined probability of being brown or moulting.

## 3 RESULTS

Between 2011 and 2019 a total of 9979 mountain hare observations were obtained at 678 camera trap locations (Figure 2 and Appendix S4). Of these observations, 7454 were recorded in ‘spring’ and 2525 were recorded in ‘autumn’. The number of camera traps deployed across Norway increased throughout the study period, leading to an increase in the number of observations obtained in each year (see Appendix S5 for location of traps that recorded observations in each year).

The results from our models indicate that all three explanatory covariates, altitude, latitude and climate zone, correlated with moult timing in spring and autumn with none of the 95% credible intervals (CI) overlapping 0. We found strong evidence for hares keeping their winter white coats for a longer duration at increased altitudes and latitudes (Figure 3a,b). The probability of being white increased with altitude in spring (*β*_2,white_ = 1.492, 95% (CI) = 1.309–1.673; Figure 3a) and in autumn (*β*_2,white_ = 0.870, 95% CI = 0.639–1.117; Figure 3a). Similarly, the probability of being white increased with latitude in spring (*β*_3,white_ = 2.472, 95% CI = 2.265–2.672; Figure 3b) and in autumn (*β*_3,white_ = 0.642, 95% CI = 0.443–0.844; Figure 3b). Climate zone had the smallest effect size in both spring and autumn. The probability of being white decreased with an increasingly inland climate (increasing PCS values) in both spring (*β*_4,white_ = −0.335, 95% CI −0.510 to −0.163; Figure 3c) and autumn (*β*_4,white_ = −0.287, 95% CI −0.525 to −0.053; Figure 3c).

The 50% probability of being white occurred 27 days earlier in spring and 23 days later in autumn at sea level, compared to 700 m above sea level (Figure 3a). Also, the 50% probability of being white occurred 27 days earlier in spring and from brown to white 14 days later in autumn at 59° N compared to 65° N (Figure 3b). Additionally, the 50% probability of being white occurred 9 days earlier in spring and 11 days later in autumn in inland climates compared to coastal climates (Figure 3c).

Mountain hare moult timing varied across mainland Norway (Figure 4). Starting in mid‐winter moving into spring, there was a travelling wave of moulting moving from lower to higher altitudes and latitudes (see Appendix S6 for animated map containing every ordinal day). The opposite effect was observed in autumn. The altitudinal and latitudinal moult timing gradients show that hares that inhabit mountainous areas and the north of Norway kept their winter white coats for longer than their conspecifics that inhabit low altitude and latitude areas. The model output indicates that mountain hares in coastal areas moult later in spring and earlier in autumn (Figure 3c). The prediction maps show that hares in southern Norway's coastal areas moult earlier in spring and later in autumn (Figure 4a,f), indicating that the effect of latitude is stronger than the effect of climate zone. This is consistent with the climate zone effect size being smaller than those of altitude and latitude. Predictions for areas outside of camera trap locations (Figure 2) should be interpreted with care as these results are extrapolated.

Timing of spring and autumn moults varied slightly between years (Figure 5). From 2013 onwards the 95% CIs are consistently narrower in spring than in autumn, which probably results from increased sample sizes in spring (Appendix S4). For the spring intercepts, multiple years have non‐overlapping CIs for 90% white, which signifies the start of spring moulting, and 90% brown, which signifies the end of spring moulting. For example, 2013 does not overlap with 2014 and 2017 does not overlap with 2018. For the autumn intercepts, the brown CIs, signifying the start of autumn moulting, overlapped in most years. The CIs for 90% white, signifying the end of autumn moulting, overlapped in all years. The time taken for moulting in spring to finish, represented by the number of days between 90% of hares being white and 90% of hares being brown, ranged between 46 days in 2019 and 64 days in 2013 and 2018 (Appendix S7). The time taken for moulting to occur in autumn ranged between 38 days in 2011 and 72 days 2013. The autumn result should be viewed with caution as 2011 and 2013 had limited sample sizes (Appendix S4).

## 4 DISCUSSION

We used camera trap by‐catch observations to provide the first quantitative assessment of how large‐scale environmental variables correlate with mountain hare moult timing. As predicted, hares at higher altitudes and latitudes moulted later, from white to brown, in spring and earlier, from brown to white, in autumn, keeping their winter white coats for longer when compared to their lowland and low latitude conspecifics. Contrary to our prediction, we found support for a slightly later spring moult and earlier autumn moult in areas characterised by coastal climates rather than inland climates, resulting in hares that live in areas with coastal climates keeping their winter white coats for longer than their inland conspecifics.

The unexpected correlation between climate zone and moult timing (Figure 3c) could result from variation explained by climate zone also being partially explained by altitude (Pearson correlation coefficient > 0.5 in both seasons) and latitude (Pearson correlation coefficient < −0.3 in both seasons; Appendix S2). Additionally, all camera traps north of 63° N are close to the coast (Figure 2) which may confound results. However, in southern Norway there is a coastal to continental moult timing gradient shown in the prediction maps (Figure 3a,f) indicating that the model has captured a correlation between moult timing and climate. Furthermore, it is likely that there will be increased variability in moult timing in coastal climates as there is increased among year variation in snow cover and duration compared to continental climates (www.senorge.no).

The altitude (Figure 3a) and latitude (Figure 3b) results are likely due to an expectation of longer snow cover duration at increased altitudes and latitudes. These results are consistent with previous studies of other mountain hare populations and other lagomorph species, which found that increased elevation correlated with mountain hares keeping their winter coats for longer (Watson, 1963) and increased latitude correlated with snowshoe hares keeping their winter white coats for longer (Grange, 1932). However, Zimova et al. (2019) found no evidence of autumn moult timing variation in snowshoe hares. Additionally, in spring, hares that lived at high latitudes moulted to brown earlier than conspecifics at lower latitudes. The correlations between moult timing and altitude and latitude indicate that mountain hare populations have adapted to local conditions, suggesting that gene flow between populations is insufficient to dilute local adaptations. This is particularly true for altitude as this variable can change significantly over a short geographic distance.

The non‐overlapping CIs between some years in the spring moult (Figure 5) indicate that there is some between year phenotypic variation. However, the between year difference in moult timing is small, which is consistent with photoperiod rather than climate being the main driver of mountain hare moult timing (reviewed in Zimova et al., 2018). This is consistent with similar studies conducted on snowshoe hares (Mills et al., 2013; Zimova et al., 2014) and least weasels (Atmeh et al., 2018), which found evidence of between year phenotypic variation in spring, but not in autumn. The limited moult timing variation may reduce fitness as an inability to change moulting patterns in response to among year variation in snow extent and duration will increase camouflage mismatch and, consequently, decrease survival probability (Zimova et al., 2016). We hypothesise that behavioural responses to mismatch, such as micro‐habitat patch selection, changed diurnal activity patterns or manually removing winter fur during the spring moult, could occur. Evidence of snowshoe hares modifying their behaviour in response to being mismatched is limited with one study suggesting that hares exhibit patch selection in response to pelage colour variation (Litvaitis, 1991) whilst one other study found no evidence of behaviour modification (Zimova et al., 2014). We are not aware of these hypotheses being investigated in mountain hares.

Moult timing variation could result from population‐level phenotypic variation, individual‐level phenotypic plasticity or a combination of both factors. As our methodology did not facilitate monitoring specific individuals, we could not disentangle the relative importance of population and individual‐level variation. Observations were obtained at specific locations in multiple years (Appendix S9). This makes it likely that some individuals were recorded in multiple years, increasing the probability of individual phenotypic plasticity influencing results. We obtained three times as many observations in spring compared to autumn (Appendix S4), which is probably caused by increased hare activity patterns during the mating season (Pettigrew et al., 2021) and the spring dataset containing observations for 58 more days. This resulted in the intercept CIs (Figure 5) being larger in autumn than in spring.

Understanding the role that environmental characteristics have on moult timing is vital when assessing the impact that climate change may have on species that express a seasonal coat colour change. Our study sites span 1300 km and 11 latitudinal degrees (58° N to 69° N), from sea level to 841 m above sea level making this is the first study that investigates moult phenology over a large, continuous climatic gradient spanning three biomes (temperate forest, boreal forest and alpine tundra). Analysing the correlation between the explanatory variables and mountain hare moult timing will enable us to predict how the species will react to climate change‐induced reductions in snow cover extent and duration. Future studies could also conduct fine‐scale analysis of the climate variables, including temperature and snow cover, that correlate with moult timing. We plan to investigate these issues in future papers.

## AUTHOR CONTRIBUTIONS

**Allan W. Stokes:** Formal analysis (lead); methodology (equal); visualization (lead); writing – original draft (lead); writing – review and editing (equal). **Tim R. Hofmeester:** Conceptualization (supporting); formal analysis (supporting); methodology (equal); visualization (supporting); writing – review and editing (equal); funding aquisition (supporting). **Neri H. Thorsen:** Formal analysis (supporting); methodology (equal); visualisation (supporting); writing – review and editing (equal). **John Odden:** Conceptualisation (supporting); methodology (equal); visualisation (supporting); Writing – review and editing (equal); funding aquisition (supporting); funding aquisition (equal). **John D. C. Linnell:** Conceptualisation (supporting); methodology (equal); visualisation (supporting); writing – review and editing (equal). **Simen Pedersen:** Conceptualization (lead); funding acquisition (lead); methodology (equal); writing – review and editing (equal).

## FUNDING INFORMATION

The study was conducted within the framework of the SCANDCAM project (https://viltkamera.nina.no), and was supported by the Norwegian Environment Agency, the Research Council of Norway (grant 281092), The Swedish Environmental Protection Agency, (Wildlife Management Fund grant NV‐2020‐00088) and the Nature Protection Division of the County Governor's Office for Innlandet, Viken, Vestfold, Telemark, Møre og Romsdal, Trøndelag, Troms and Finnmark Counties.

## CONFLICT OF INTEREST STATEMENT

We confirm that there are no conflicts of interest.

## PERMISSION TO REPRODUCE MATERIALS

None.

## Supporting information


Appendix S1.

## Data Availability

The dataset used for analysis including phenotypic and environmental data and the location where the observations were collected: Figshare, DOI: 10.6084/m9.figshare.22560340.
