# Supplementary material for: Altitude, latitude and climate zone as determinants of mountain hare (Lepus timidus) coat colour change
Source: Ecol Evol. 2023 Oct 1;13(10):e10548. doi: 10.1002/ece3.10548 (PMC10542609; doi:10.1002/ece3.10548)
Supplement: Supplementary file 1 — Appendix S1. [file ECE3-13-e10548-s001.zip › Suppl figures combined.pdf]

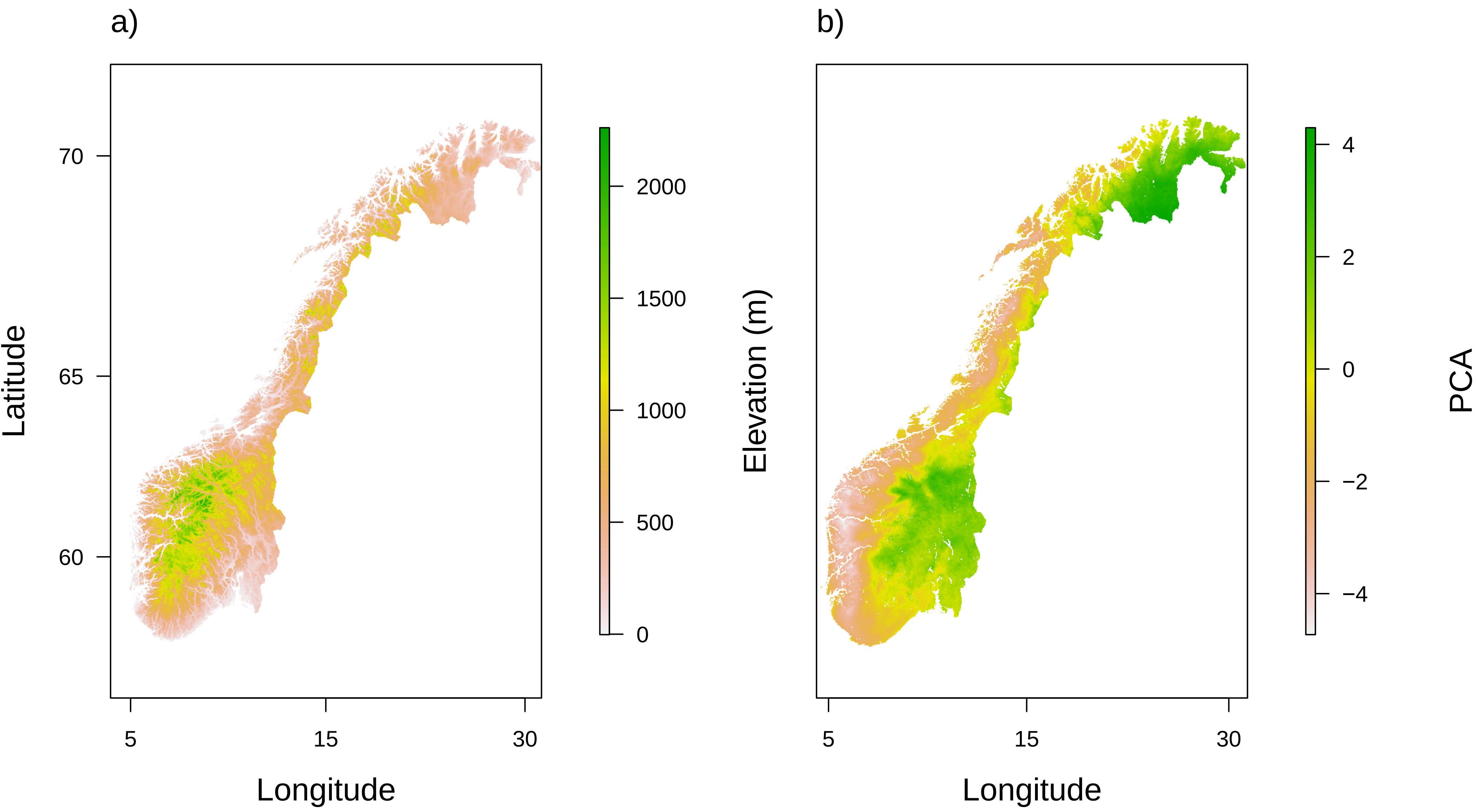
a)
b)
70
65
60
Latitude
5
15
30
Longitude
2000
1500
1000
500
0
Elevation (m)
4
2
0
−2
−4
PCA

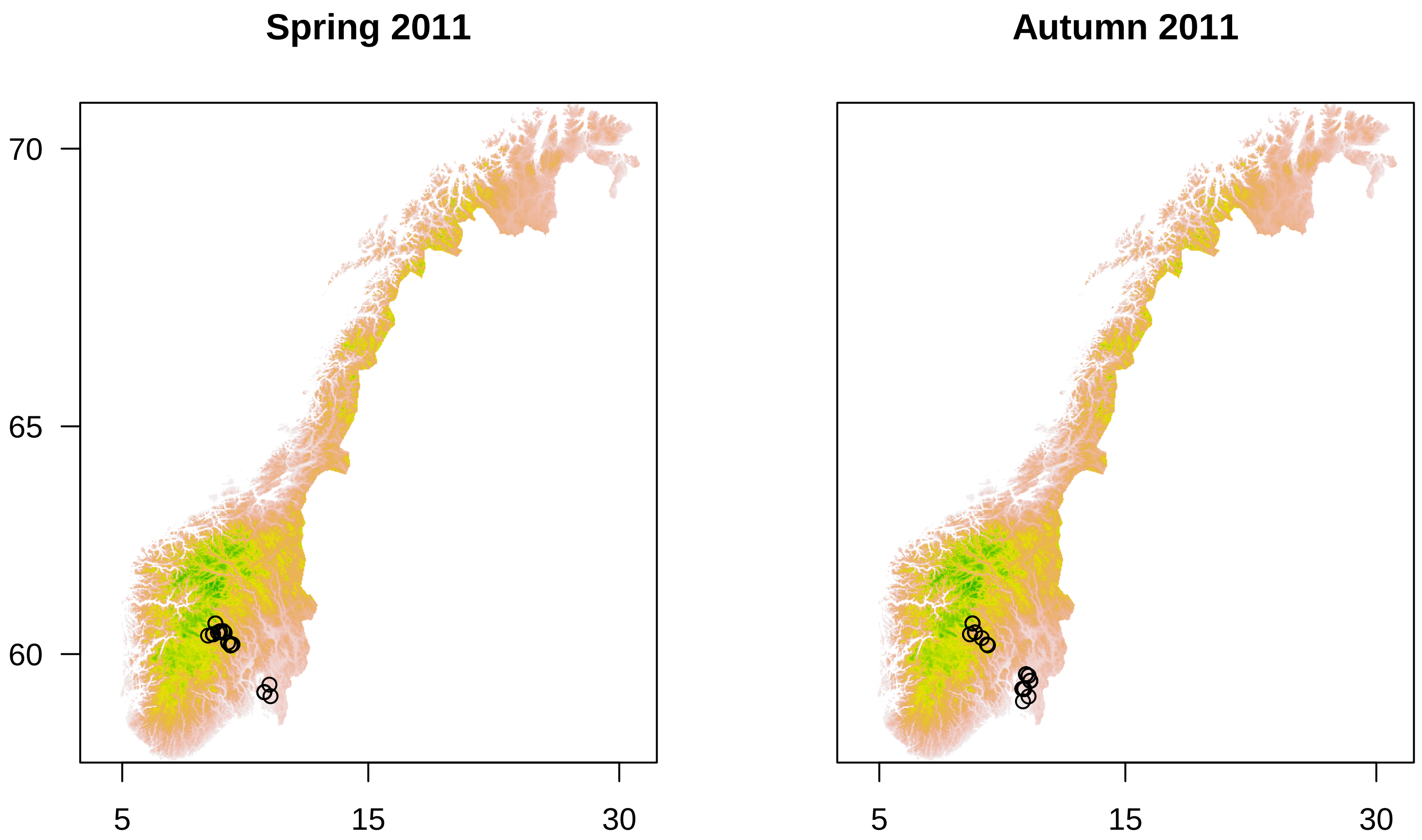
Spring 2011
Autumn 2011
70
65
60
5
15
30

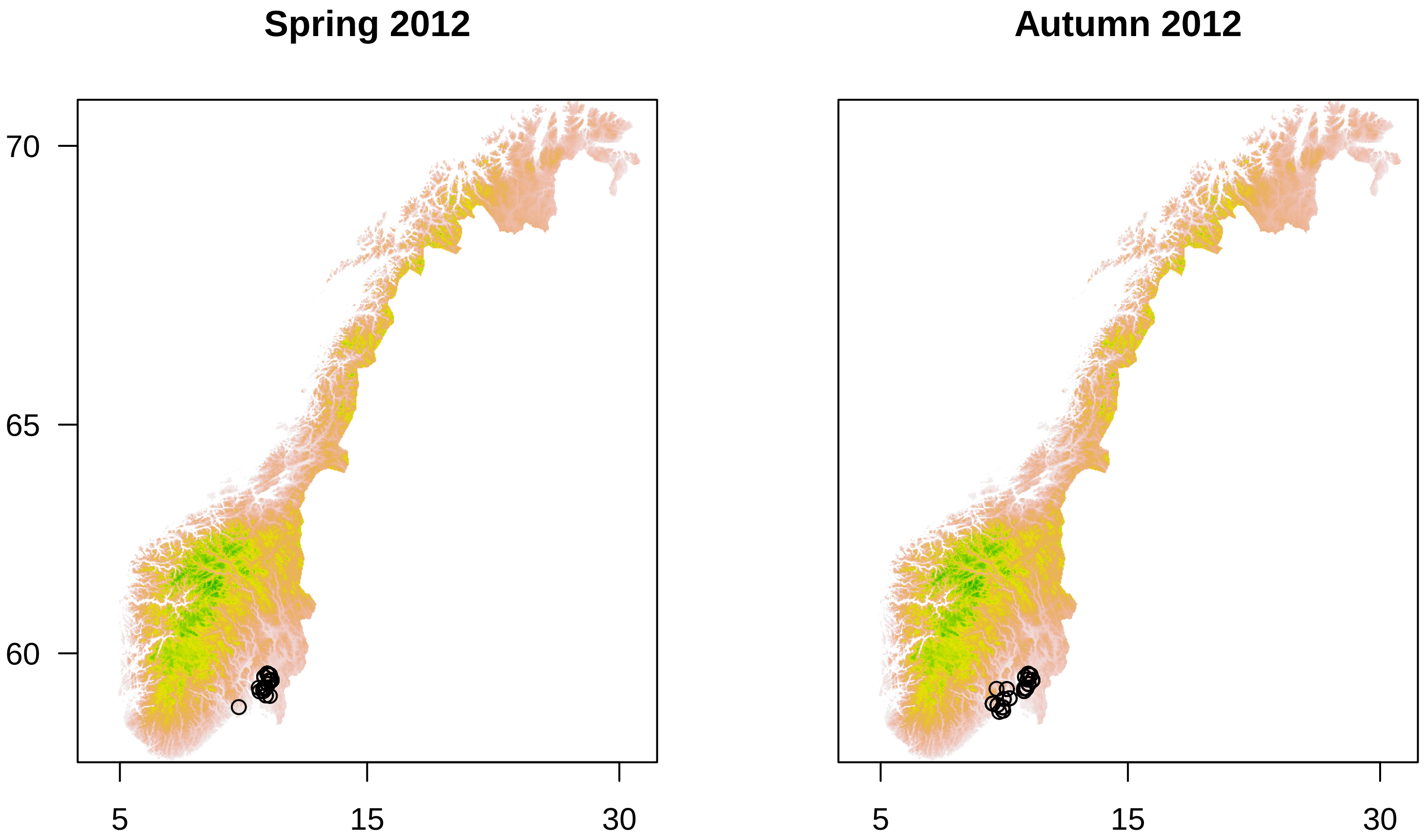
Spring 2012
Autumn 2012
70
65
60
5
15
30
5
15
30

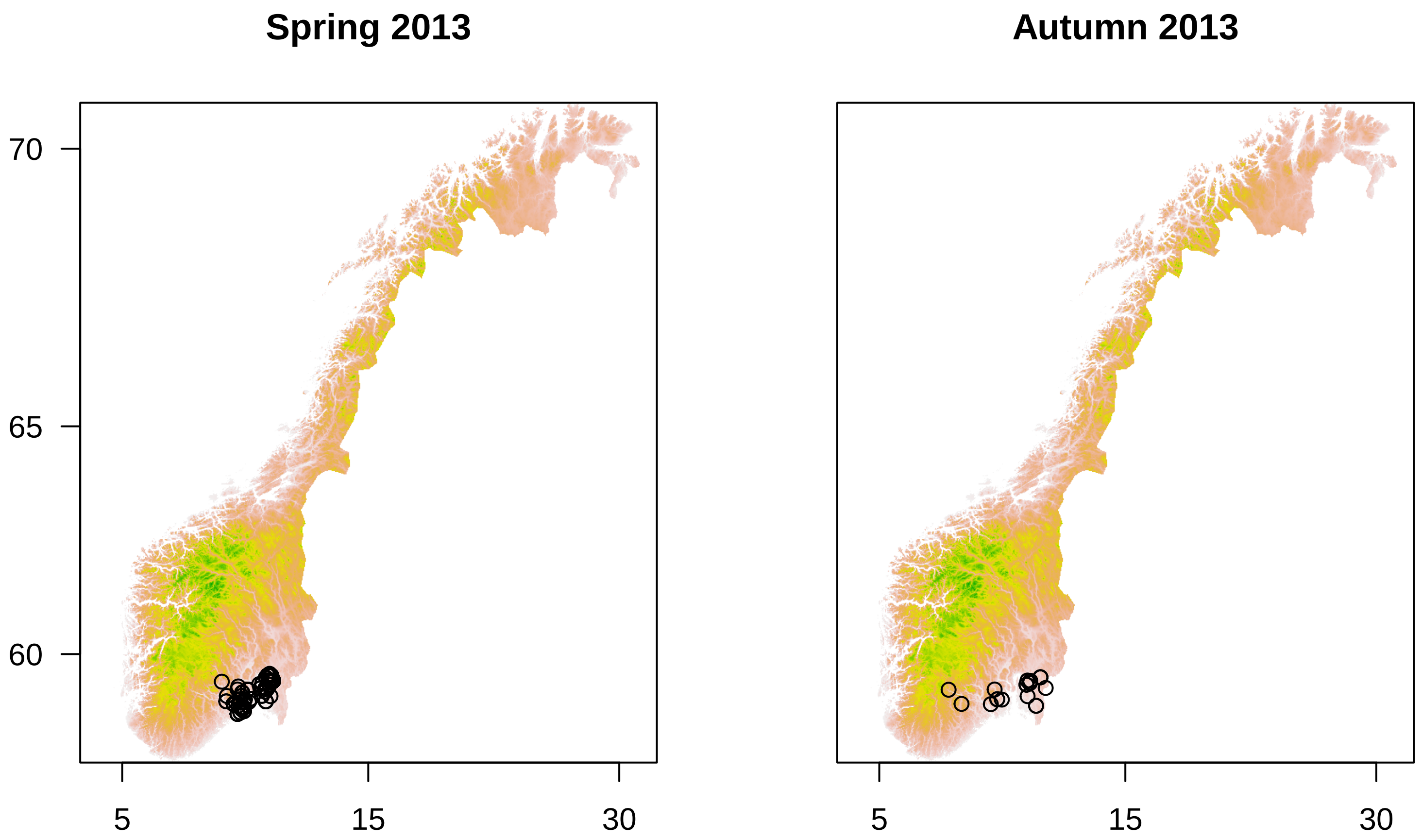
Spring 2013
Autumn 2013
70
65
60
5
15
30
5
15
30

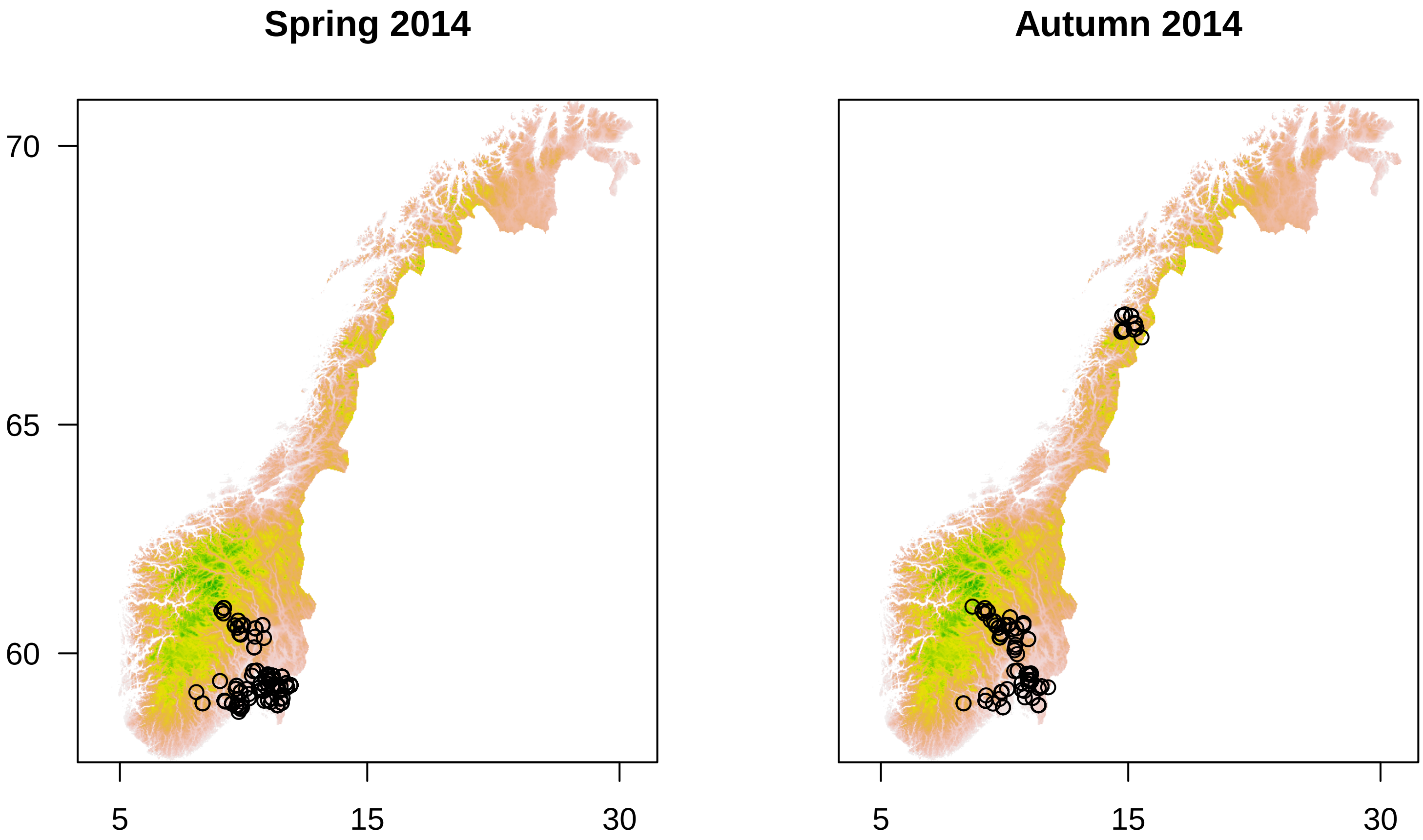
Spring 2014
Autumn 2014
70
65
60
5
15
30
5
15
30

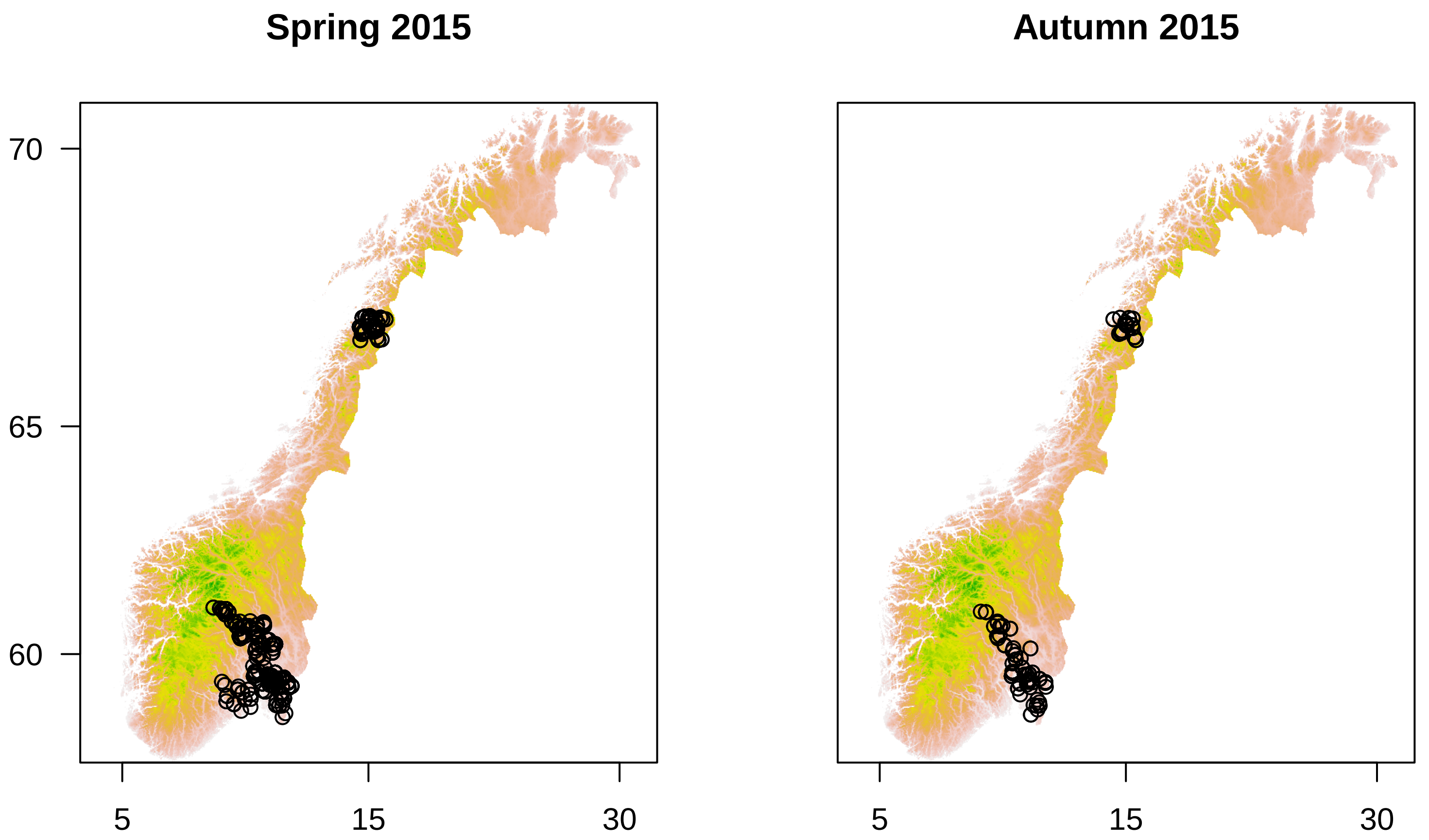

Spring 2015
Autumn 2015
70
65
60
5
15
30
5
15
30

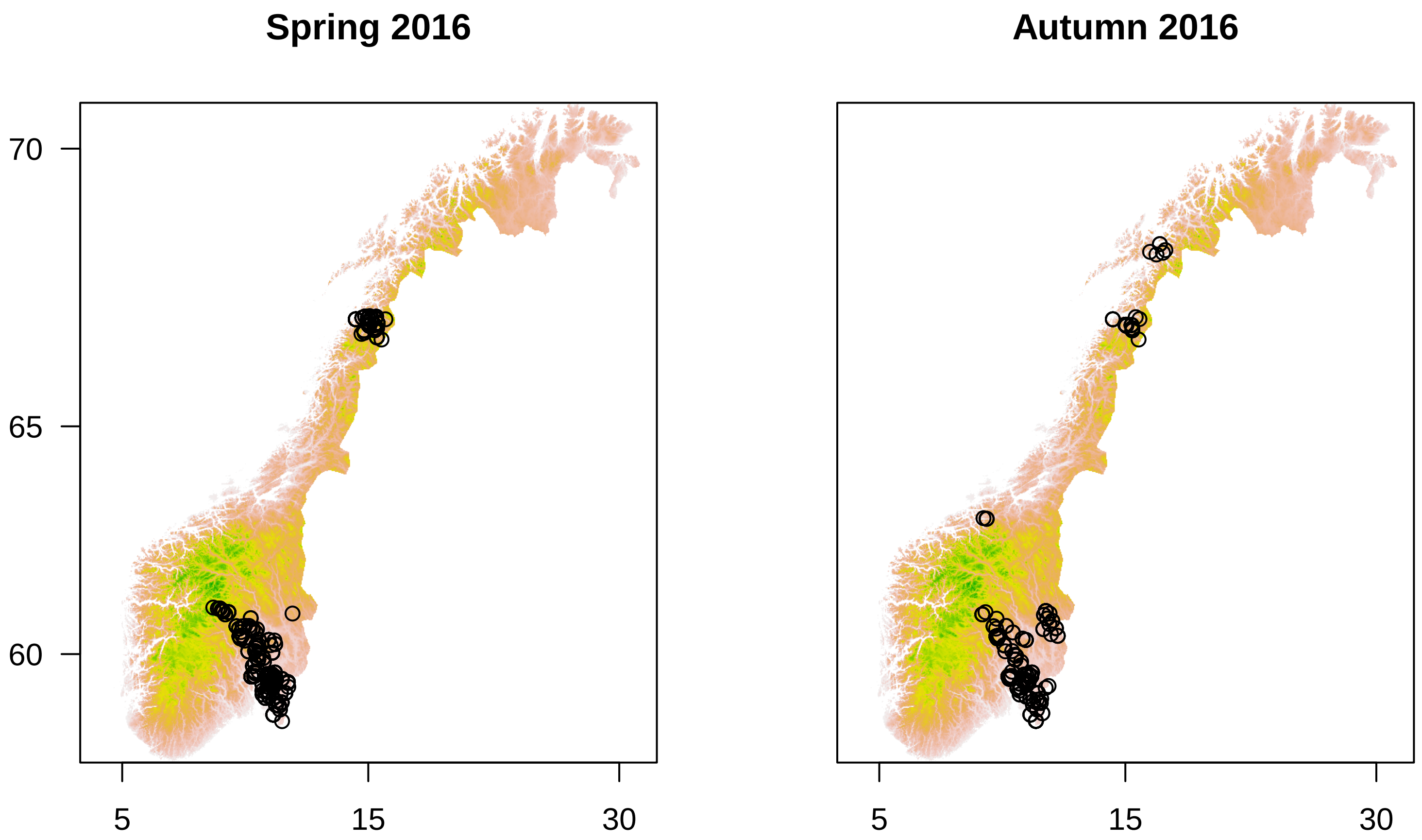
Spring 2016
Autumn 2016
70
65
60
5
15
30
5
15
30

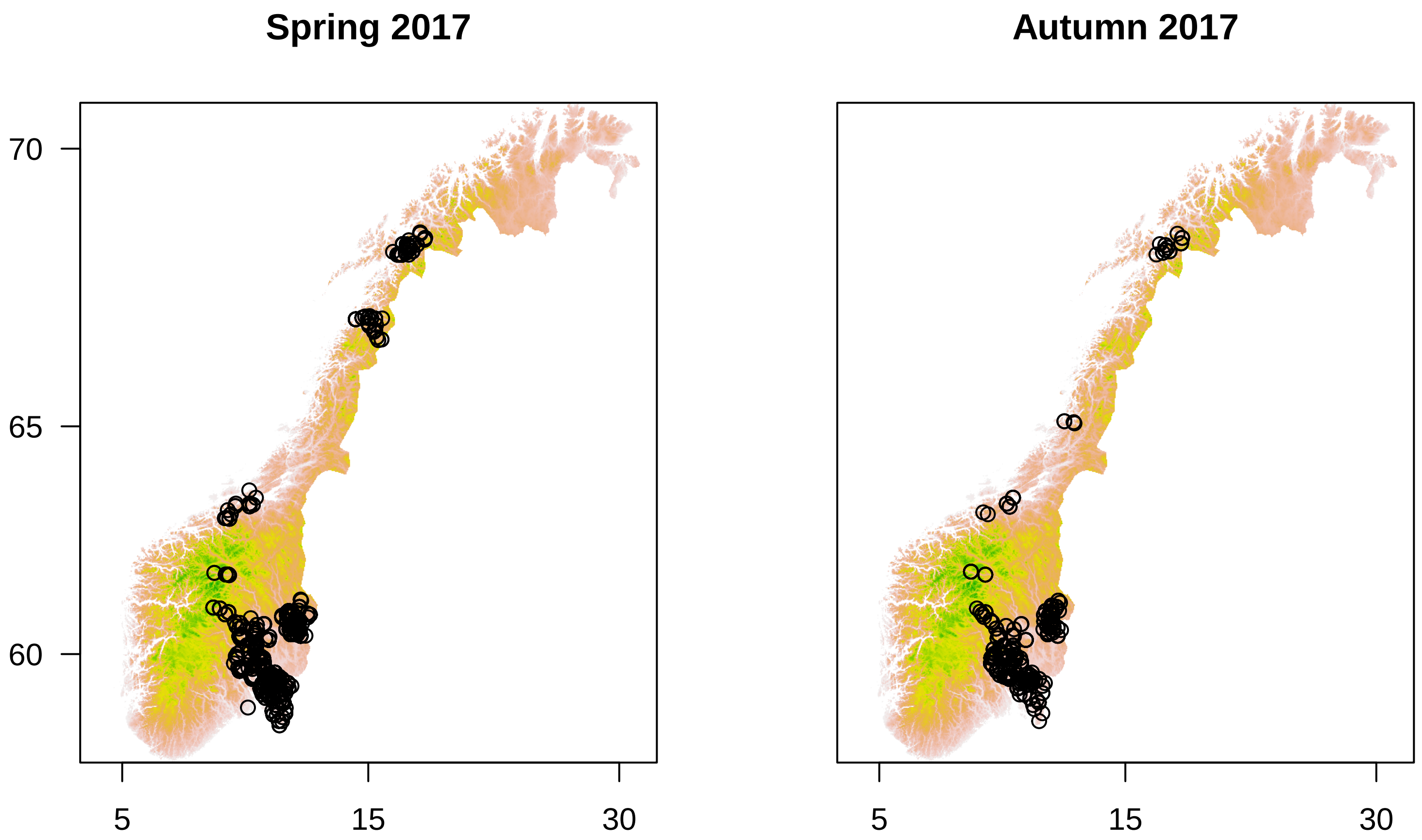
Spring 2017
Autumn 2017
70
65
60
5
15
30
5
15
30

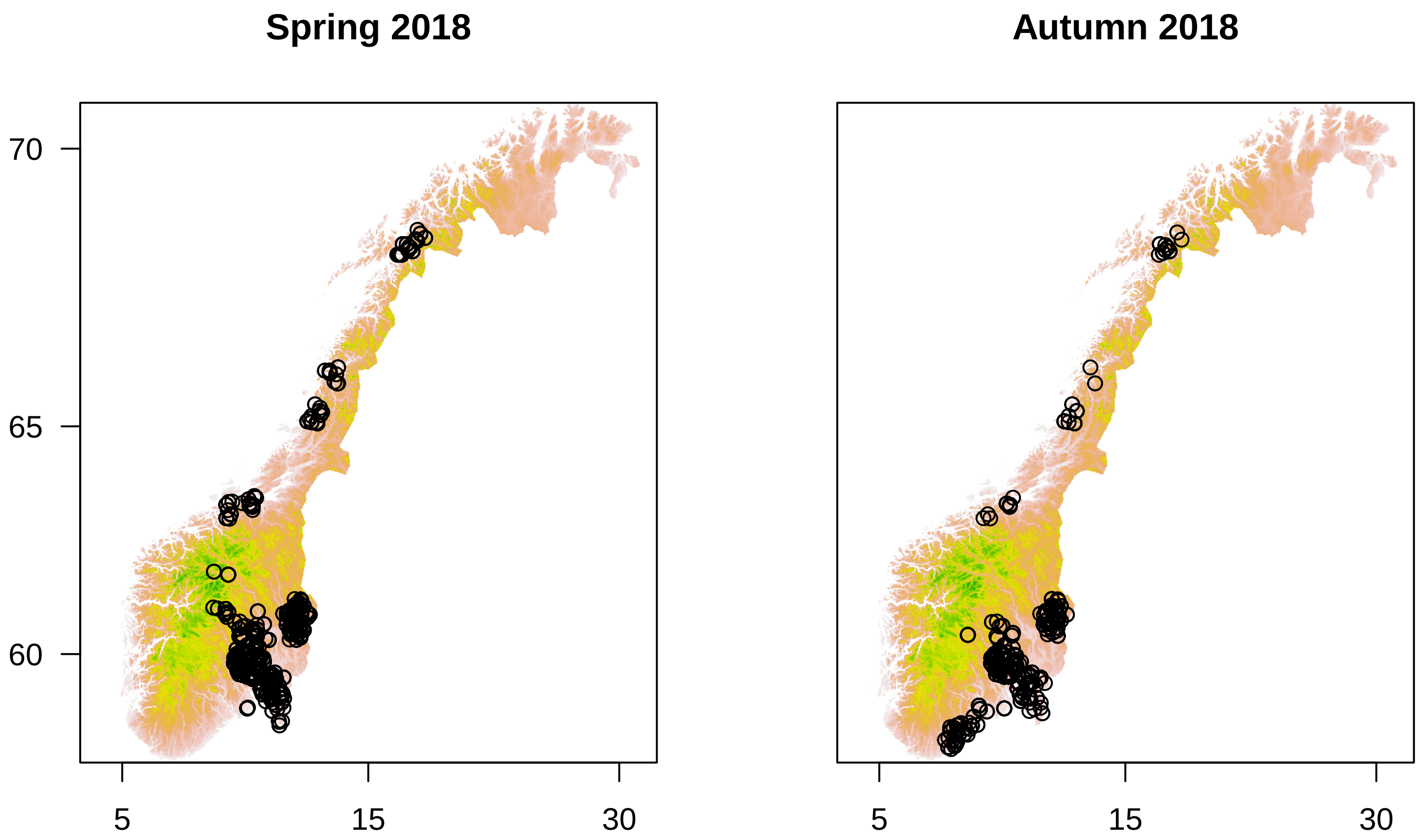
Spring 2018
Autumn 2018
70
65
60
5
15
30

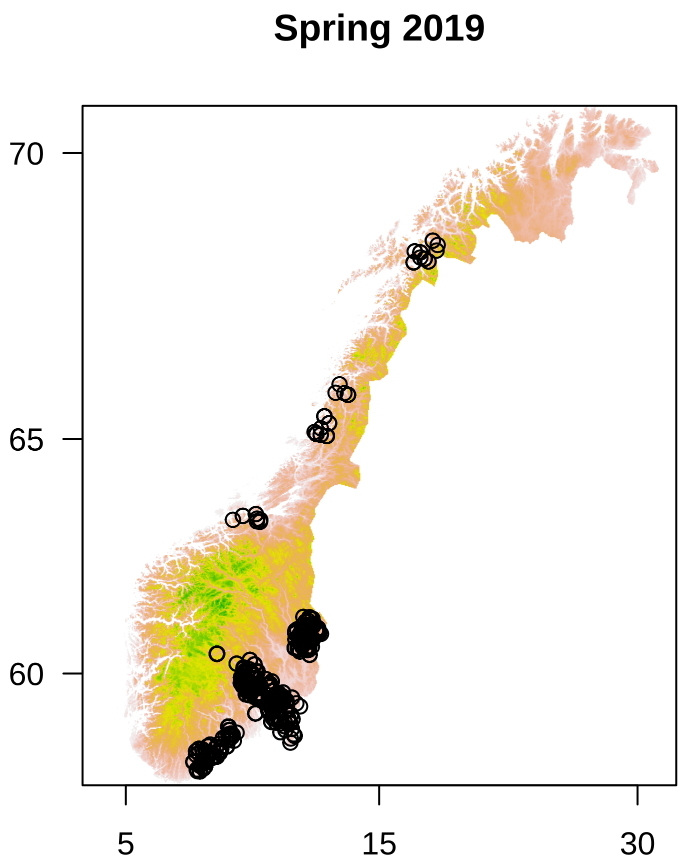

Spring 2019
70
65
60
5
15
30
