# Supplementary material for: Altitude, latitude and climate zone as determinants of mountain hare (Lepus timidus) coat colour change
Source: Ecol Evol. 2023 Oct 1;13(10):e10548. doi: 10.1002/ece3.10548 (PMC10542609; doi:10.1002/ece3.10548)
Supplement: Supplementary file 1 — Appendix S1. [file ECE3-13-e10548-s001.zip › Supplementary captions.docx]

Suppl 6. Prediction map animation with a resolution of 1 x 1 km2 giving the probability of mountain hares being white across Norway on every day of the year. The probability of being white was predicted using the environmental covariates contained in each cell and the model output.
