# Supplementary material for: Altitude, latitude and climate zone as determinants of mountain hare (Lepus timidus) coat colour change
Source: Ecol Evol. 2023 Oct 1;13(10):e10548. doi: 10.1002/ece3.10548 (PMC10542609; doi:10.1002/ece3.10548)
Supplement: Supplementary file 1 — Appendix S1. [file ECE3-13-e10548-s001.zip › Suppl_data.docx]

Supplemental information


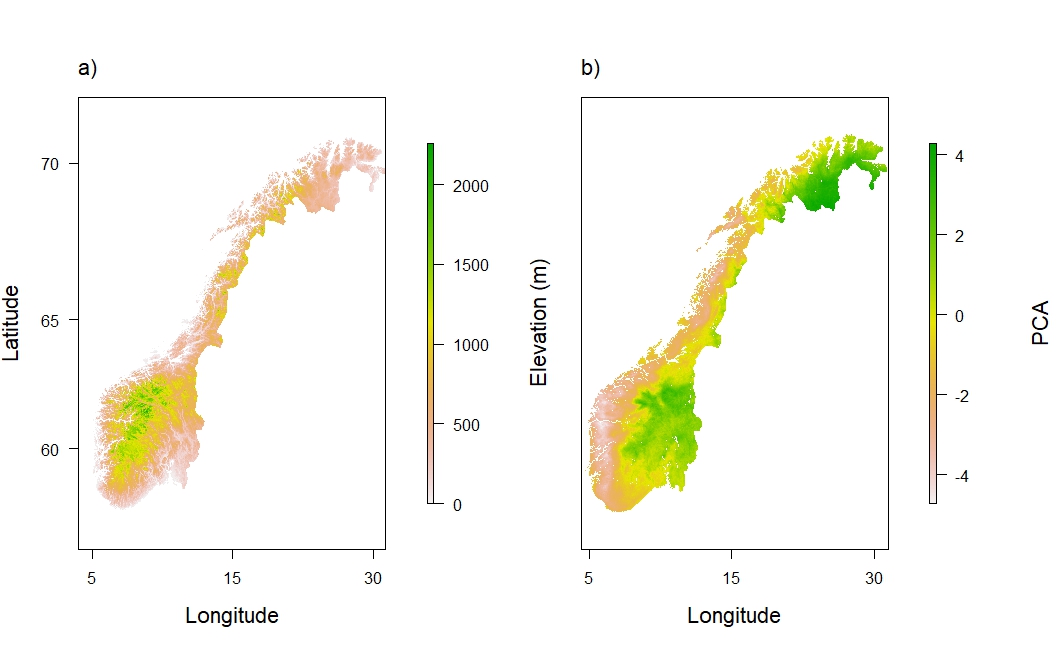


Suppl 1.a) Digital elevation model (Kartkatalogen 2007) with a resolution of 50 m^2^ giving the altitude across Norway. The altitude of the camera traps used in this study varies between 0 m and 841 m above sea level. b) Climatic zones across Norway obtained from Bakkestuen et al (2008). Negative values indicate an oceanic climate whereas positive values indicate a continental climate. Values were obtained via principle component analysis (PCA).

Suppl 2. Pearson’s correlation coefficient (3 SF) between altitude, latitude and climate zone in spring and autumn. All years and camera sites were used for the calculations. The datasets were subset so that only one observation per site was used for the calculation.

| Spring | | | Autumn | | |
| --- | --- | --- | --- | --- | --- |
| Altitude * latitude | Altitude * climate zone | Latitude * climate zone | Altitude * latitude | Altitude * climate zone | Latitude * climate zone |
| -0.0295 | 0.530 | -0.362 | -0.0649 | 0.548 | -0.306 |

Suppl 3. Variance inflation factor (VIF) (3 SF) between elevation, latitude and climate zone in spring and autumn. All years and camera sites were used for the calculations. The datasets were subset so that only one observation per site was used for the calculation.

| Spring | | | Autumn | | |
| --- | --- | --- | --- | --- | --- |
| Altitude | Latitude | Climate zone | Altitude | Latitude | Climate zone |
| 1.47 | 1.20 | 1.69 | 1.45 | 1.12 | 1.60 |

Suppl 4. The number of samples recorded by camera traps in spring and autumn from 2011 to 2019. The number of samples recorded increased as the study progressed due to an increase in the number of traps deployed.

| Year | Spring | Autumn |
| --- | --- | --- |
| 2011 | 40 | 36 |
| 2012 | 113 | 80 |
| 2013 | 258 | 23 |
| 2014 | 308 | 165 |
| 2015 | 859 | 204 |
| 2016 | 602 | 358 |
| 2017 | 1,517 | 644 |
| 2018 | 2,035 | 1,027 |
| 2019 | 1,738 | NA |


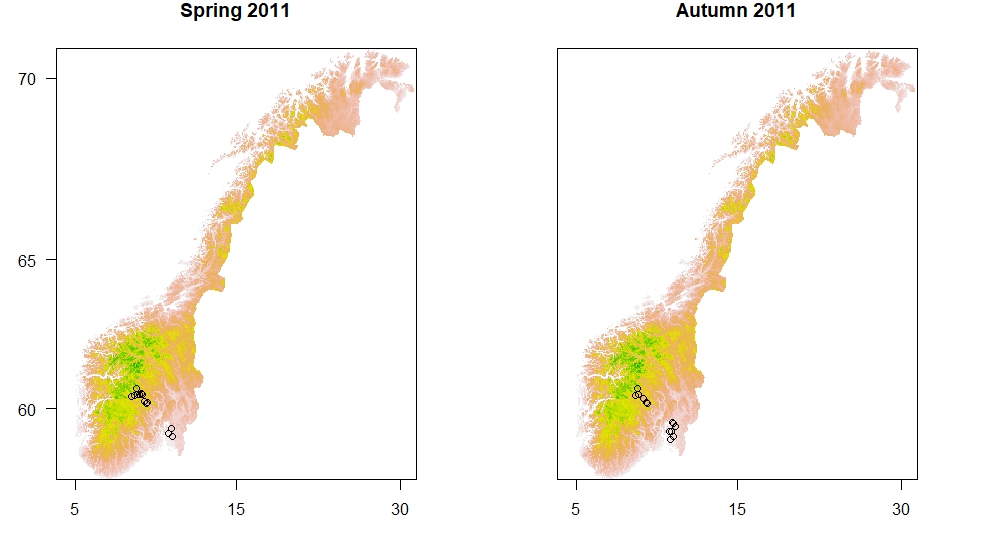


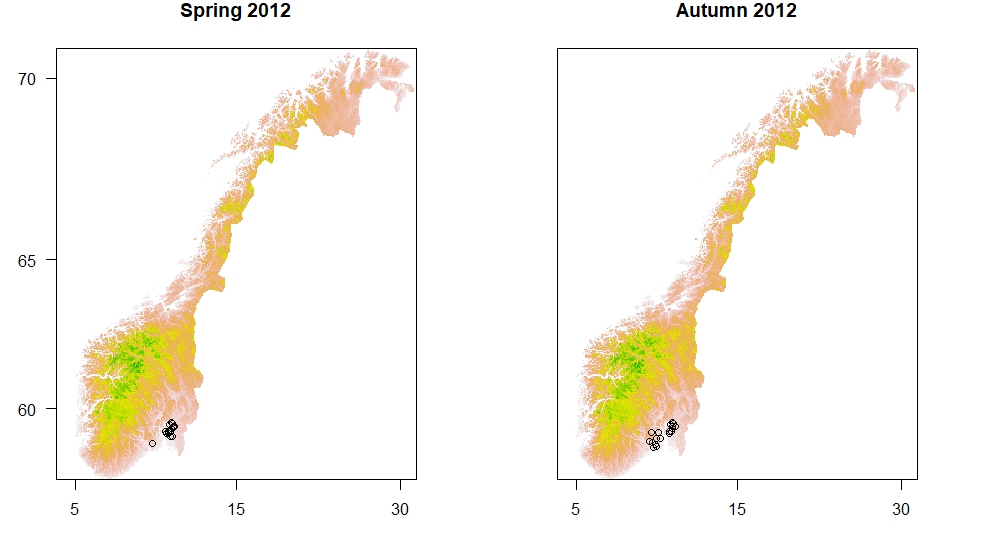


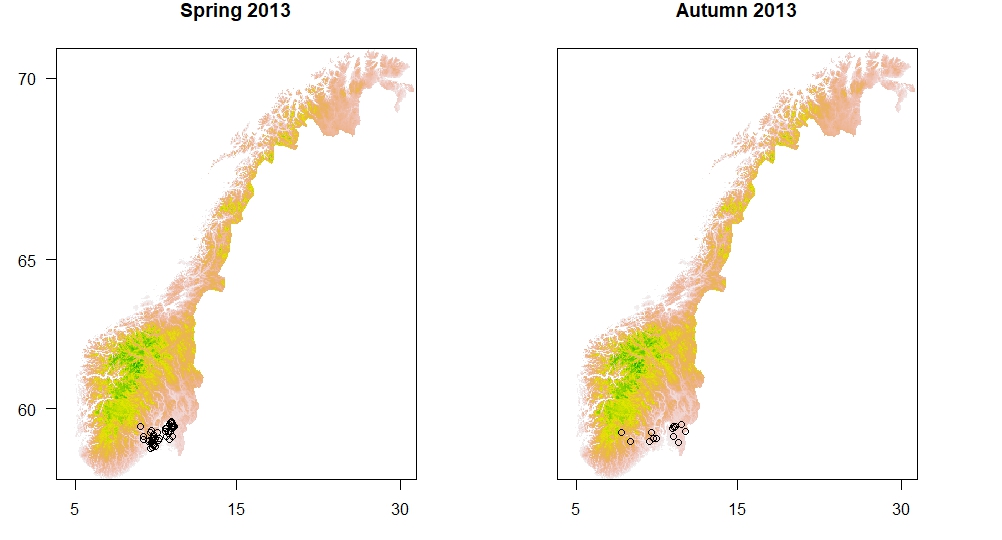

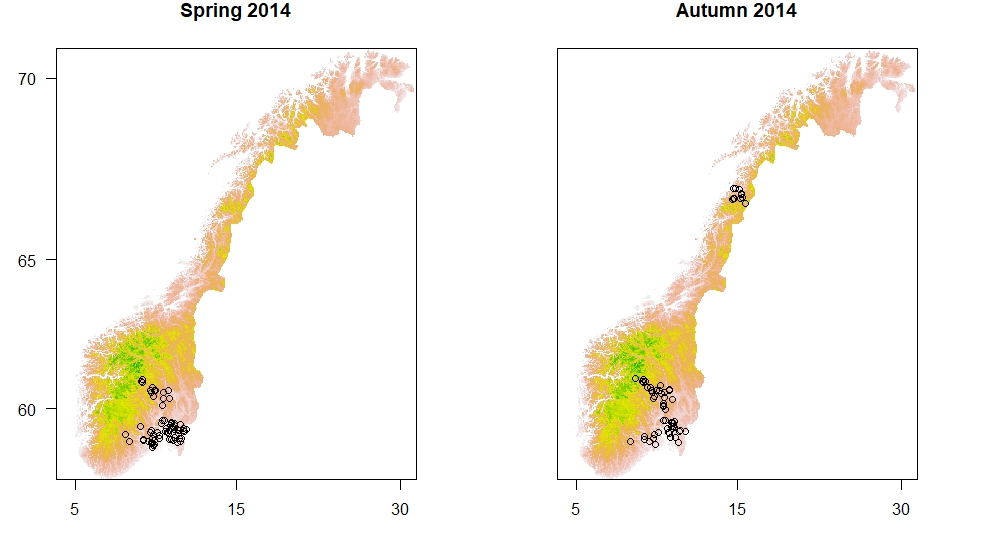

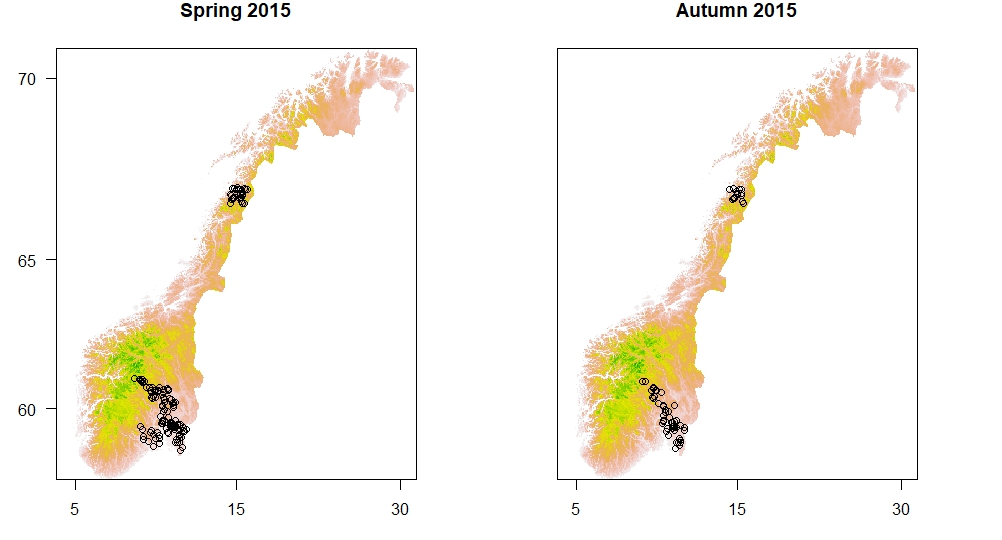


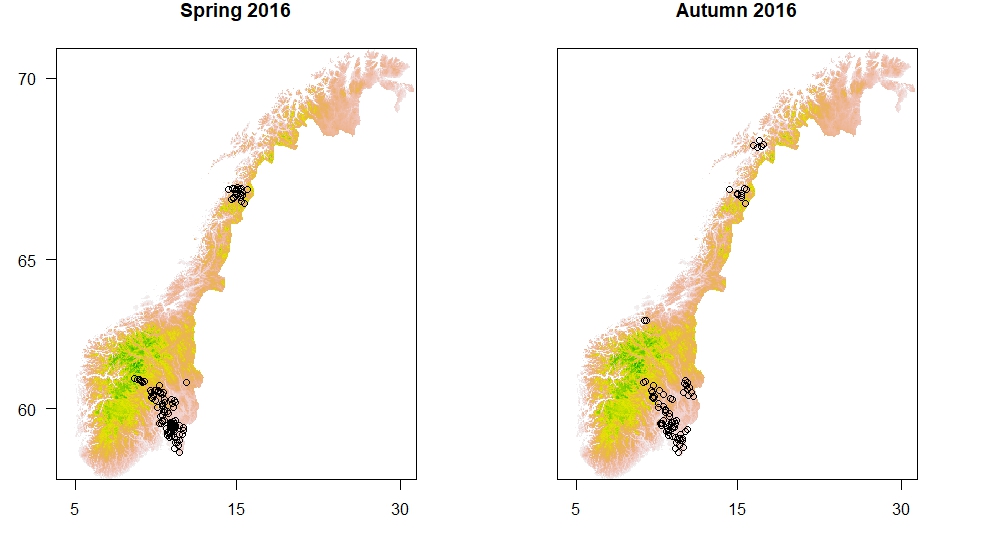


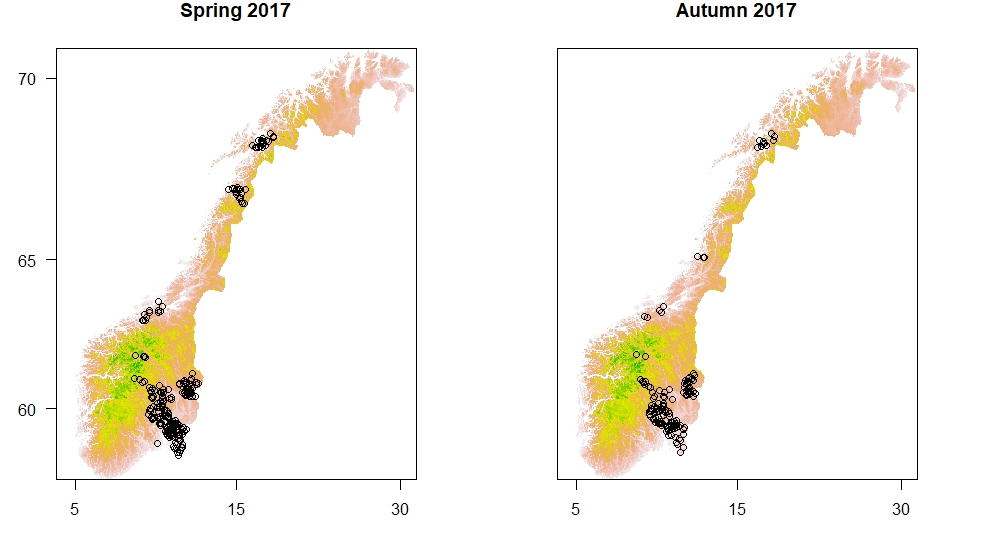


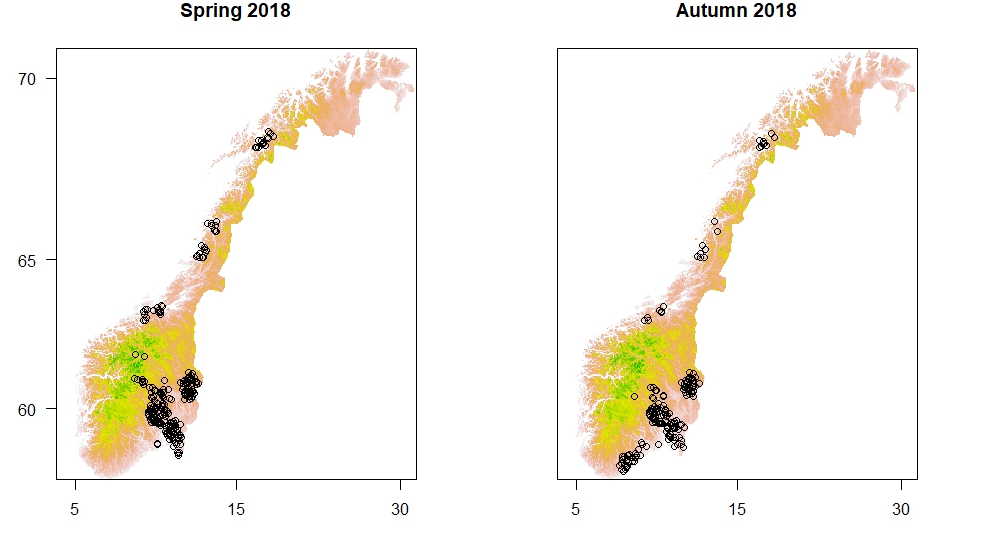


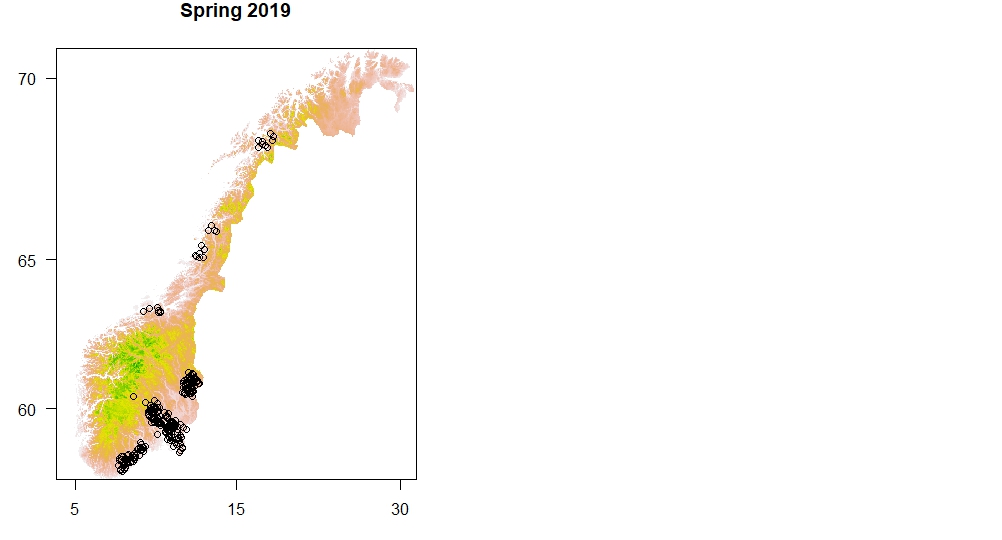


Supp 5. Location of camera traps that recorded at least one observation in spring and autumn from spring 2011 to spring 2019. The camera trap locations are laid over the DEM (Suppl 1).


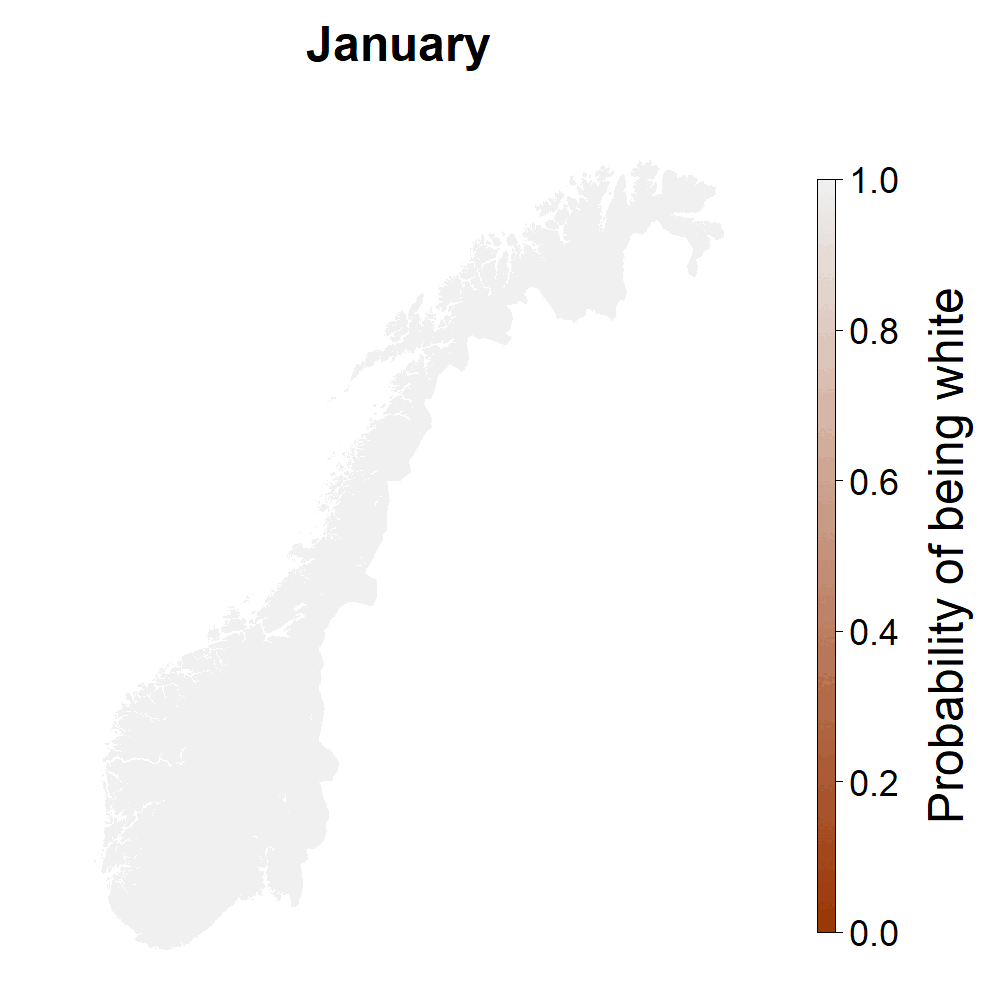


Suppl 6. Prediction map animation with a resolution of 1 x 1 km^2^ giving the probability of mountain hares being white across Norway on every day of the year. The probability of being white was predicted using the environmental covariates contained in each cell and the model output.

Suppl 7. The ordinal days in which moult was initiated and finished in spring and autumn. In spring, moult initiation was defined as the day on which less than 90% of the hare was white and moult end was defined as the day on which less than 90% of the hare was white. In autumn, moult initiation was defined as the day on which less than 90% of the hare was brown and moult end was defined as the day on which less than 90% of the hare was brown.

| Year | Ordinal day when spring moult started | Ordinal day when spring moult finished | Days taken to moult in spring | Ordinal day when autumn moult started | Ordinal day when autumn moult finished | Days taken to moult in autumn |
| --- | --- | --- | --- | --- | --- | --- |
| 2011 | 74 | 124 | 50 | 292 | 330 | 38 |
| 2012 | 75 | 135 | 60 | 278 | 329 | 51 |
| 2013 | 101 | 165 | 64 | 262 | 334 | 72 |
| 2014 | 88 | 142 | 54 | 266 | 326 | 60 |
| 2015 | 90 | 141 | 51 | 273 | 326 | 53 |
| 2016 | 92 | 143 | 51 | 284 | 332 | 48 |
| 2017 | 84 | 136 | 52 | 278 | 331 | 53 |
| 2018 | 100 | 150 | 50 | 281 | 332 | 51 |
| 2019 | 96 | 142 | 46 | NA | NA | NA |

Suppl 8. The beta intercepts taken from our Bayesian multinomial logistic regression model. They illustrate the between season and year variation in moult timing.

| Year | Spring (95% CI) | Autumn (95% CI) |
| --- | --- | --- |
| 2011 | 20.932 (15.809 to 29.746) | -44.525 (-48.253 to -40.601) |
| 2012 | 21.438 (19.727 to 23.146) | -43.931 (-47.367 to -40.340) |
| 2013 | 27.432 (25.804 to 29.129) | -43.677 (-47.693 to -39.776) |
| 2014 | 23.819 (22.364 to 25.148) | -43.201 (-46.692 to -39.672) |
| 2015 | 23.923 (22.530 to 25.338) | -43.494 (-46.964 to -39.944) |
| 2016 | 24.338 (22.925 to 25.727) | -44.456 (-47.803 to -40.869) |
| 2017 | 22.761 (21.478 to 24.114) | -44.174 (-47.477 to -40.675) |
| 2018 | 26.066 (24.622 to 27.529) | -44.424 (-47.751 to -40.946) |
| 2019 | 24.942 (23.549 to 26.324) | NA |

Suppl 9. The number of years that observations were obtained in for every camera trap location that obtained a mountain hare observation.

| Number of camera trap locations | Number of years samples were obtained in |
| --- | --- |
| 304 | 1 |
| 180 | 2 |
| 116 | 3 |
| 39 | 4 |
| 30 | 5 |
| 4 | 6 |
| 1 | 7 |
| 2 | 8 |
| 2 | 9 |
